# Estimation bias in survival data within clinical trials that use adaptive seamless designs

**DOI:** 10.1186/1745-6215-16-S2-P220

**Published:** 2015-11-16

**Authors:** Josephine Khan, Peter Kimani, Nigel Stallard, Ekkehard Glimm

**Affiliations:** 1 University of Warwick, Coventry, UK; 2 Novartis Pharma AG, Basel, Switzerland

## 

Adaptive designs are utilised in late phase clinical trials due to their efficiency in answering multiple clinical questions through a single trial. For example, seamless phase II/III clinical trials are used to answer phase II and III objectives via a single trial with two stages, where stage 1 and 2 represent phase II and III components respectively. At the end of stage1, an interim analysis is performed to make treatment selection, a phase II objective. At the end of stage 2, data from both stages are used to perform a confirmatory analysis, a phase III objective. Although efficient, data dependent selection introduces complexity in estimation.

For normally distributed outcomes, unbiased point estimators for phase II/III trials have been developed. In this work, we focus on survival data, with treatment effect quantified by the log hazard ratio (log(HR)). Using asymptotic theory, with no selection, the log-rank statistic divided by the information is normally distributed, with mean equal to the true log(HR). Although we can assume normality, survival outcomes have an additional complexity of censoring. Patients who do not experience the event at the time of interim-analysis are censored and then followed further in stage 2. This induces correlation between stage 1 and 2 data. We will firstly present the range of true log(HR) for which the normality assumption works well, whilst illustrating the bias introduced by both treatment selection and censoring at the interim-analysis. We will then describe the progress in addressing the challenge of correlated stage 1 and 2 data.

